# Supplementary figures and images for: Candida albicans white and opaque cells exhibit distinct spectra of organ colonization in mouse models of infection
Source: PLoS One. 2019 Jun 6;14(6):e0218037. doi: 10.1371/journal.pone.0218037 (PMC6553767; doi:10.1371/journal.pone.0218037)

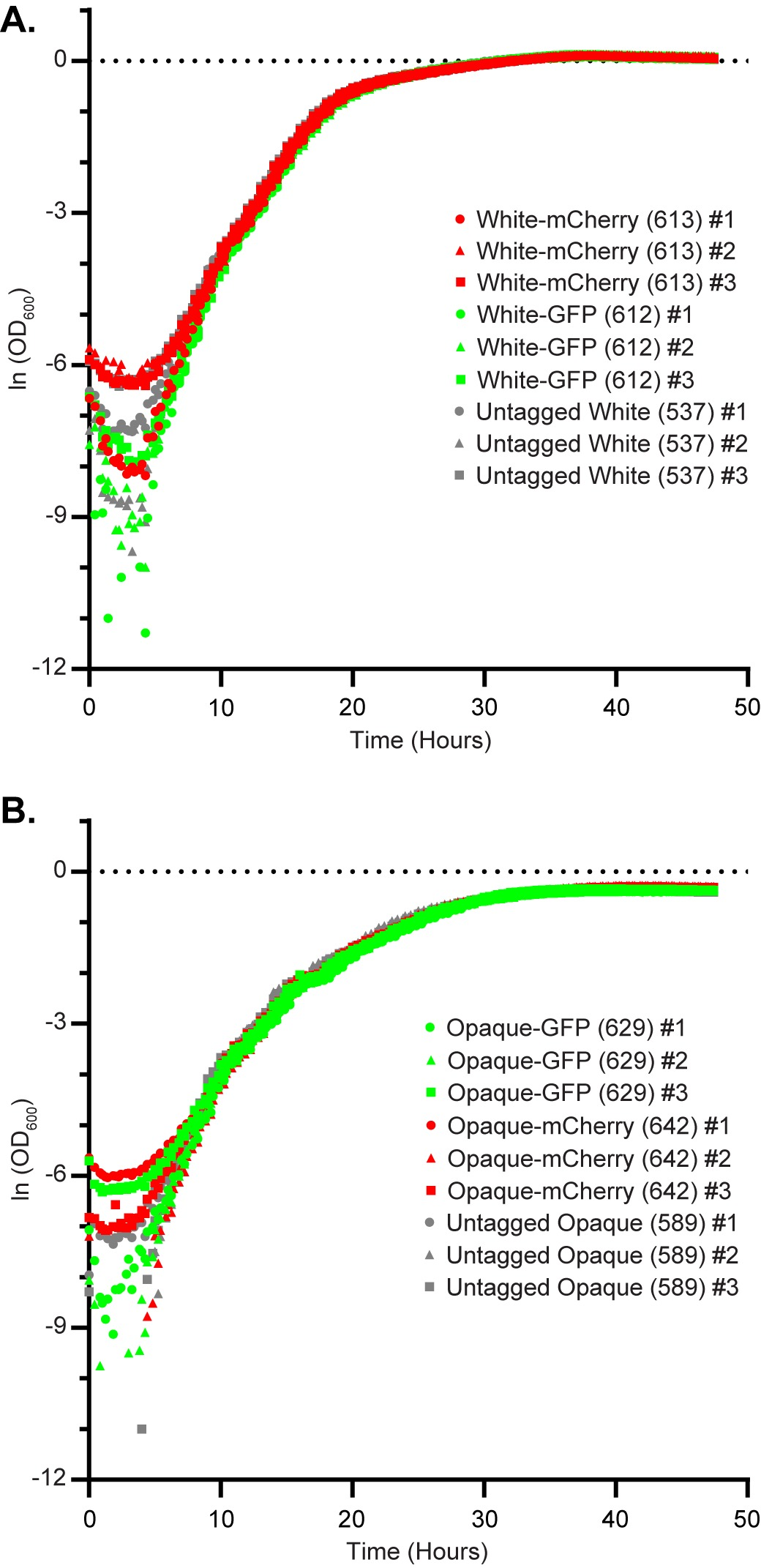

Supplement: S1 Fig — (a) White cells without a fluorescent tag (grey), white cells expressing GFP (green), and white cells expressing mCherry (red) were grown in SD+AA+Uri at 25°C. (b) Opaque cells without a fluorescent tag (grey), opaque cells expressing GFP (green), and opaque cells expressing mCherry (red) were grown in SD+AA+Uri at 25°C. In both panels the natural logarithm of the background subtracted absorbance at 600 nm, averaged from three technical replicates, is plotted as a function of time for the three biological replicates of each strain. (TIF) [file pone.0218037.s001.tif]

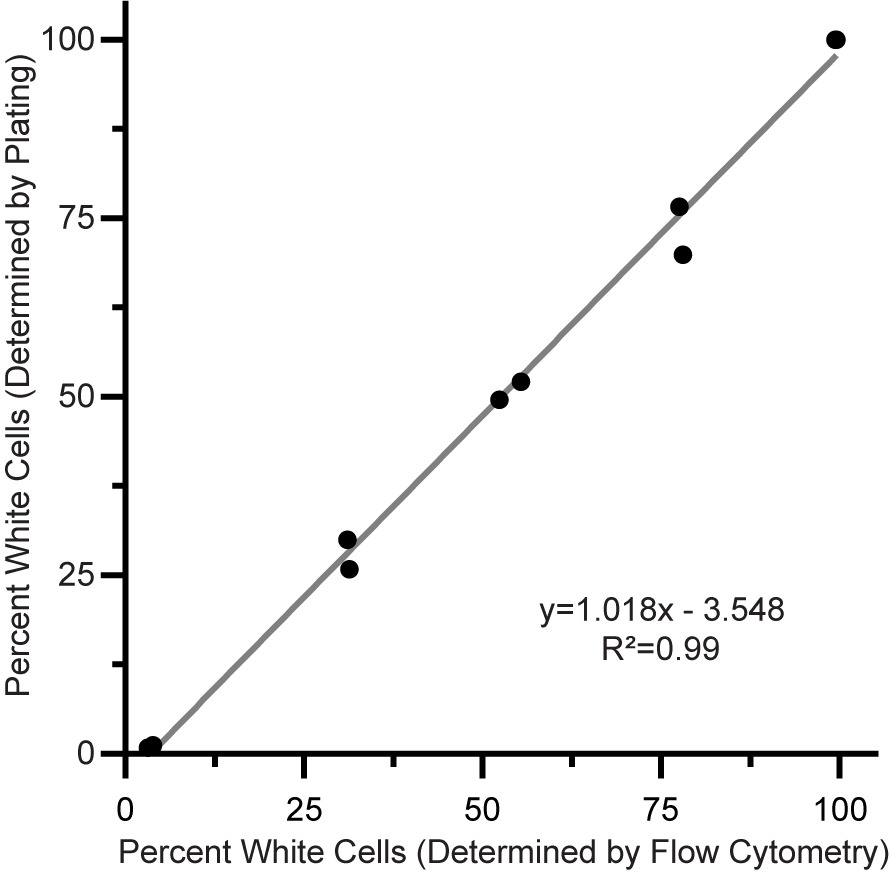

Supplement: S2 Fig — Opaque-GFP and white-mCherry cultures were independently grown. The cell density and fraction of the population expressing GFP (that is, opaque cells) or not expressing GFP (that is, white cells) were independently measured using a flow cytometer. The white and opaque cultures were then mixed at different ratios and the fraction of the population not expressing GFP (that is, white cells) was determined for each mixture using a flow cytometer. The mixtures were then plated and subsequently scored for colony phenotype. The proportion of the culture that did not express GFP (that is, white cells) was compared to the proportion of white colonies. The linear regression is indicated in grey. (TIF) [file pone.0218037.s002.tif]

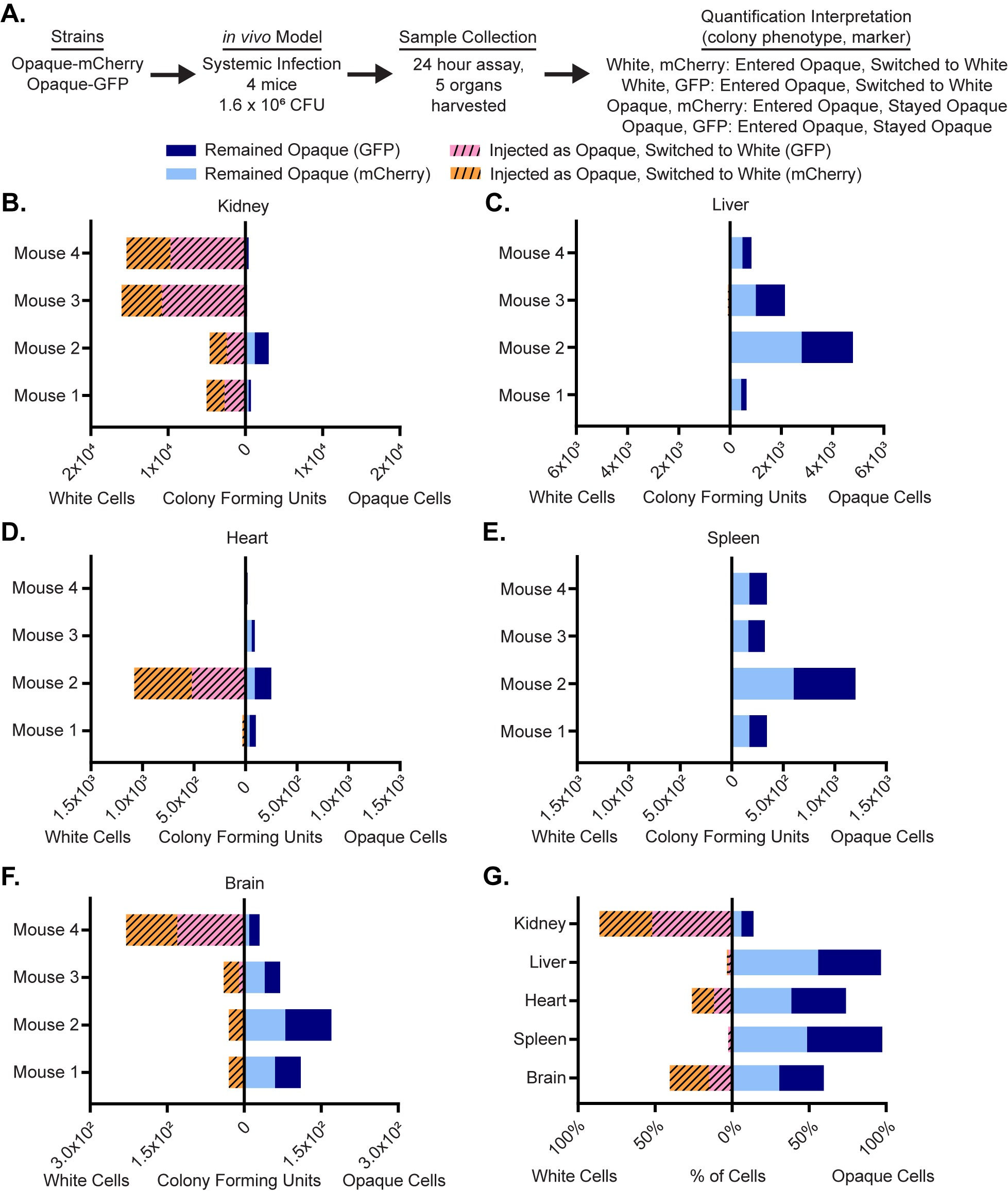

Supplement: S3 Fig — (a) Using a flowchart, the experimental setup, cell type, and potential fluorescence phenotypes for each strain are tabulated. In this case, opaque cells expressing mCherry and opaque cells expressing GFP were co-injected into the tail-veins of 4 mice. Five organs, the kidney, liver, heart, spleen and brain, were processed to measure white and opaque cell colonization as well as white-opaque switching. The mechanistic interpretation of each phenotype; in other words, whether or not it indicates cell-type switching, is also indicated. The colony-forming units of cells that remained opaque (i.e. opaque cells expressing mCherry (light blue) or GFP (blue)) and of cells that switched from opaque-to-white (i.e. white cells expressing GFP (dashed pink) or mCherry (dashed orange)) are plotted for each mouse for the (b) kidney, (c) liver, (d) heart, (e) spleen and (f) brain. The left side of each horizontal bar graph refers to cells that were white at the end of the experiment while the right side of each horizontal bar graph refers to cells that were opaque at the end of the experiment. (g) The mean percentage of total cells that remained opaque (i.e. opaque cells expressing mCherry (light blue) or GFP (blue)) or that switched from opaque-to-white (i.e. white cells expressing GFP (dashed pink) or mCherry (dashed orange)) are plotted per organ as a horizontal bar graph. The left side of the horizontal bar graph refers to cells that were white at the end of the experiment while the right side of the horizontal bar graph refers to cells that were opaque at the end of the experiment. The raw data for this experiment is available in S4 Data. (TIF) [file pone.0218037.s003.tif]

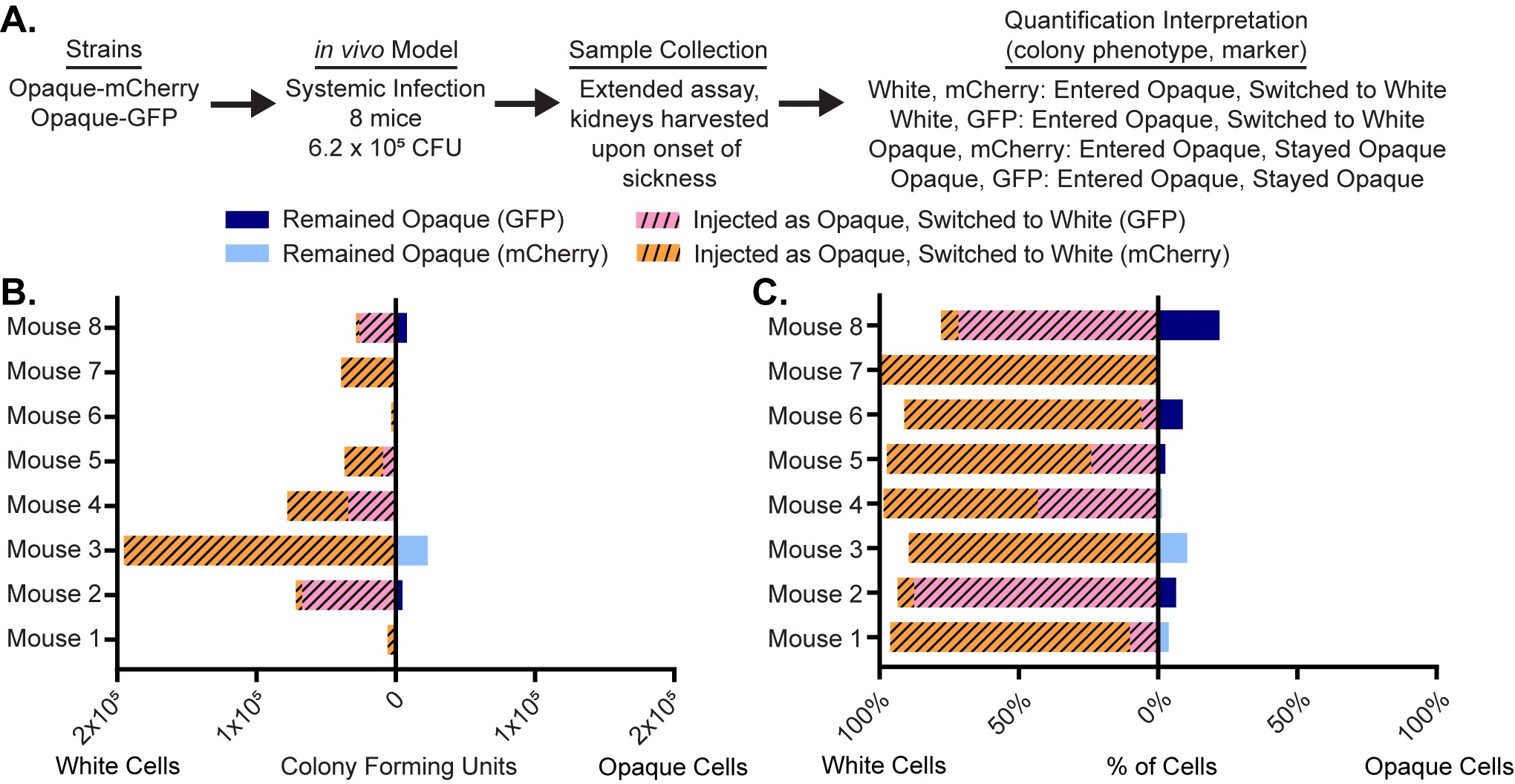

Supplement: S4 Fig — (a) Using a flowchart, the experimental setup, cell type, and potential fluorescence phenotypes for each strain are tabulated. In this case, opaque cells expressing mCherry and opaque cells expressing GFP were co-injected into the tail-veins of 8 mice. Upon the onset of illness, the kidney was processed to measure opaque cell colonization as well as opaque-to-white switching. The mechanistic interpretation of each phenotype; in other words, whether or not it indicates cell-type switching, is also indicated. (b) The colony-forming units of cells that remained opaque (i.e. opaque cells expressing mCherry (light blue) or GFP (blue)) and of cells that switched from opaque-to-white (i.e. white cells expressing GFP (dashed pink) or mCherry (dashed orange)) are plotted per mouse as a horizontal bar graph. The left side of the horizontal bar graph refers to cells that were white at the end of the experiment while the right side of the horizontal bar graph refers to cells that were opaque at the end of the experiment. (c) The percentage of total cells that remained opaque (i.e. opaque cells expressing mCherry (light blue) or GFP (blue)) or that switched from opaque-to-white (i.e. white cells expressing GFP (dashed pink) or mCherry (dashed orange)) are plotted for each mouse as a horizontal bar graph. The left side of the horizontal bar graph refers to cells that were white at the end of the experiment while the right side of the horizontal bar graph refers to cells that were opaque at the end of the experiment. The raw data for this experiment is available in S5 Data. (TIF) [file pone.0218037.s004.tif]

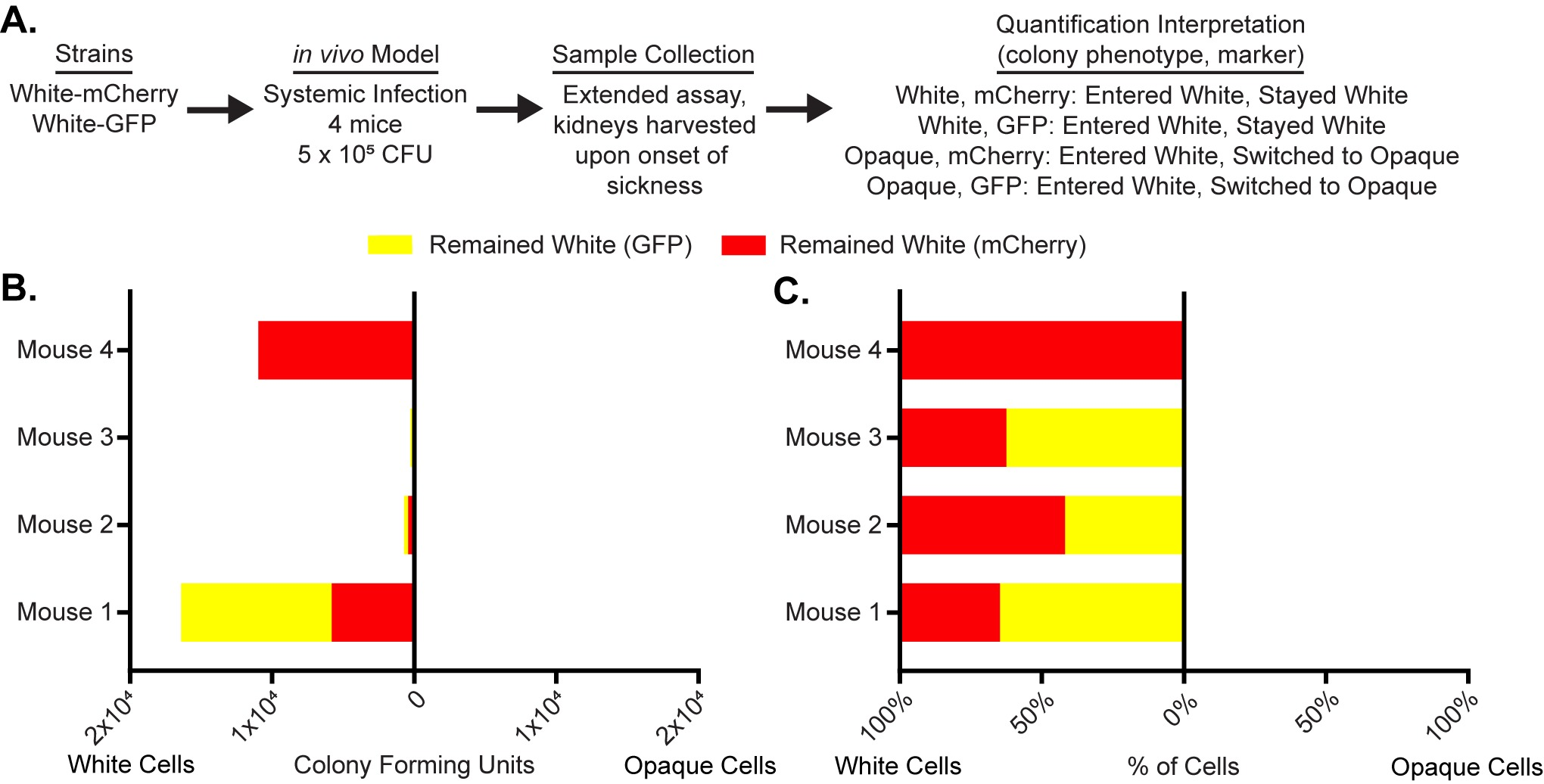

Supplement: S5 Fig — (a) Using a flowchart, the experimental setup, cell type, and potential fluorescence phenotypes for each strain are tabulated. In this case, white cells expressing mCherry and white cells expressing GFP were co-injected into the tail-veins of 4 mice. Upon the onset of illness, the kidney was processed to measure white cell colonization as well as white-opaque switching. The mechanistic interpretation of each phenotype; in other words, whether or not it indicates cell-type switching, is also indicated. (b) The colony-forming units of white cells expressing GFP (yellow) and the colony-forming units of white cells expressing mCherry (red) are plotted per mouse as a horizontal bar graph. The left side of the horizontal bar graph refers to cells that were white at the end of the experiment while the right side of the horizontal bar graph refers to cells that were opaque at the end of the experiment. (c) The percentage of total cells that remained white (i.e. white cells expressing mCherry (red) or GFP (yellow)) are plotted for each mouse as a horizontal bar graph. The left side of the horizontal bar graph refers to cells that were white at the end of the experiment while the right side of the horizontal bar graph refers to cells that were opaque at the end of the experiment. The raw data for this experiment is available in S5 Data. (TIF) [file pone.0218037.s005.tif]
